# Automated assessment of endometrial receptivity for screening recurrent pregnancy loss risk using deep learning-enhanced ultrasound and clinical data

**DOI:** 10.3389/fphys.2024.1404418

**Published:** 2024-12-24

**Authors:** Shanling Yan, Fei Xiong, Yanfen Xin, Zhuyu Zhou, Wanqing Liu

**Affiliations:** ^1^ Department of Ultrasound, Deyang People’s Hospital, Deyang, Sichuan, China; ^2^ Department of Obstetrics and Gynecology, Deyang People’s Hospital, Deyang, Sichuan, China

**Keywords:** recurrent pregnancy loss, endometrial receptivity, deep learning, nomogram, ultrasound, routine examination

## Abstract

**Background:**

Recurrent pregnancy loss (RPL) poses significant challenges in clinical management due to an unclear etiology in over half the cases. Traditional screening methods, including ultrasonographic evaluation of endometrial receptivity (ER), have been debated for their efficacy in identifying high-risk individuals. Despite the potential of artificial intelligence, notably deep learning (DL), to enhance medical imaging analysis, its application in ER assessment for RPL risk stratification remains underexplored.

**Objective:**

This study aims to leverage DL techniques in the analysis of routine clinical and ultrasound examination data to refine ER assessment within RPL management.

**Methods:**

Employing a retrospective, controlled design, this study included 346 individuals with unexplained RPL and 369 controls to assess ER. Participants were allocated into training (n = 485) and testing (n = 230) datasets for model construction and performance evaluation, respectively. DL techniques were applied to analyze conventional grayscale ultrasound images and clinical data, utilizing a pre-trained ResNet-50 model for imaging analysis and TabNet for tabular data interpretation. The model outputs were calibrated to generate probabilistic scores, representing the risk of RPL. Both comparative analyses and ablation studies were performed using ResNet-50, TabNet, and a combined fusion model. These were evaluated against other state-of-the-art DL and machine learning (ML) models, with the results validated against the testing dataset.

**Results:**

The comparative analysis demonstrated that the ResNet-50 model outperformed other DL architectures, achieving the highest accuracy and the lowest Brier score. Similarly, the TabNet model exceeded the performance of traditional ML models. Ablation studies demonstrated that the fusion model, which integrates both data modalities and is presented through a nomogram, provided the most accurate predictions, with an area under the curve of 0.853. The radiological DL model made a more significant contribution to the overall performance of the fusion model, underscoring its superior predictive capability.

**Conclusion:**

This investigation demonstrates the superiority of a DL-enhanced fusion model that integrates routine ultrasound and clinical data for accurate stratification of RPL risk, offering significant advancements over traditional methods.

## Introduction

Recurrent pregnancy loss (RPL) remains a contentious issue due to the lack of universally accepted guidelines, leading to ongoing debates about clinical management, diagnostic protocols, and potential interventions. According to the latest guidelines from the European Society of Human Reproduction and Embryology (ESHRE), RPL is defined as two or more miscarriages before the 24th week of gestation (TEGGo et al., 2023). This represents a shift from the previous definition, which required three or more miscarriages, as upheld by both ESHRE and the Royal College of Obstetrics and Gynecology (No RG-tG, 2011). It is anticipated that this redefinition may significantly increase the recognized prevalence of RPL, which currently affects an estimated 1%–5% of women, suggesting that the true incidence may be higher.

Addressing the challenges in RPL management requires a strategy focused on clinical risk screening to precisely identify at-risk individuals. This typically involves identifying known and potential risk factors and examining their correlation with the onset of RPL (Turesheva et al., 2023). Despite substantial clinical efforts to clarify factors such as infections, metabolic disorders, chromosomal, endocrine, immunological irregularities, and uterine anatomical abnormalities as established causes of RPL, more than half of affected couples lack a clear cause for pregnancy failure (Cao et al., 2022). This lack of identifiable reasons complicates the assessment of RPL risk.

Given these insights, our strategy seeks to refine the RPL screening process by extensively utilizing ultrasound imaging. RPL patients often exhibit a condition known as “superfertility”, evidenced by inadequate fibroblast decidualization and an uncoordinated maternal response to embryonic signals (Teklenburg et al., 2010). This condition is speculated to extend the window of endometrial receptivity (ER), potentially delaying the implantation of compromised embryos (Patel and Lessey, 2011). As supported by Wilcox et al. (1999), evaluating ER differences between women with RPL and healthy individuals could be crucial for identifying those at higher risk. Recent studies on ultrasonographic ER evaluation have mainly highlighted endometrial features crucial for predicting outcomes in assisted reproductive technologies, such as endometrial morphology and Doppler blood flow (Khan et al., 2016; Bahrami et al., 2023; Sharfi et al., 2015). However, the utility of these features in identifying RPL risk remains debated.

With the evolution of information technology, digital image analysis in assessing ER has reached new levels of precision, even integrating advanced techniques such as radiomics (Fournier et al., 2021; Huang et al., 2023). However, the complexity and time-consuming nature of these methods limit their routine clinical adoption as screening tools. Recent advancements have highlighted the significant role of deep learning (DL) technologies, especially convolutional neural networks (CNNs), in enhancing the utility of medical imaging data (Iglesias et al., 2021). Celebrated for their capabilities in image recognition and classification, CNNs present a promising path for managing the complex, high-dimensional data intrinsic to ultrasound imaging (Cheng and Malhi, 2017). This facilitates the identification of intricate patterns associated with ER states, offering a sophisticated, computer-aided method for ER evaluation. DL-enhanced ER assessments are expected to improve diagnostic accuracy for RPL while minimizing additional workload for clinicians. Initial efforts have demonstrated that integrating DL with clinical data and ultrasound can effectively predict pregnancy outcomes and aid in diagnosis (Liang et al., 2023; Wang et al., 2022). Despite these advancements, the application of DL innovations in specifically stratifying RPL risk has not been thoroughly explored, leaving a significant gap in the current research.

This study presents a novel approach by employing DL techniques for analyzing routine clinical and ultrasound data. Unlike traditional methods that rely on three-dimensional imaging, our approach focuses on leveraging DL to extract and interpret subtle features from routine diagnostic evaluations. This innovation enhances the precision of ER assessments and offers a more efficient and accessible method for managing RPL risk. By integrating ultrasound data and clinical parameters, this research proposes an improved methodology for RPL risk stratification, offering significant clinical value. Ultimately, our approach is expected to lead to the development of personalized management strategies for RPL patients, improving prognostic outcomes and optimizing clinical decision-making.

## Material and methods

This investigation was conducted in accordance with a retrospective, controlled design and adhered strictly to ethical standards as prescribed by the Declaration of Helsinki. Approval for the study was granted by the Institutional Review Board of Deyang People’s Hospital (2022-04-083-K01). Owing to its retrospective design, the ethical committee provided a waiver for the requirement of informed consent from participants. Measures were taken to anonymize participant data thoroughly prior to analysis, safeguarding their confidentiality and privacy.

### Participants

From 2021 to 2023, this study compiled data from transvaginal ultrasounds and routine clinical examinations of 400 patients suffering from unexplained RPL (uRPL), defined as women who had experienced two or more consecutive pregnancy losses without identifiable causes such as autoimmune, anatomical, genetic, endocrine, infectious, or male factor issues (Yu et al., 2023). Additionally, 400 women who had undergone thorough fertility assessments and subsequently achieved full-term pregnancies without previous losses were included as the control cohort.

**FIGURE 1:**
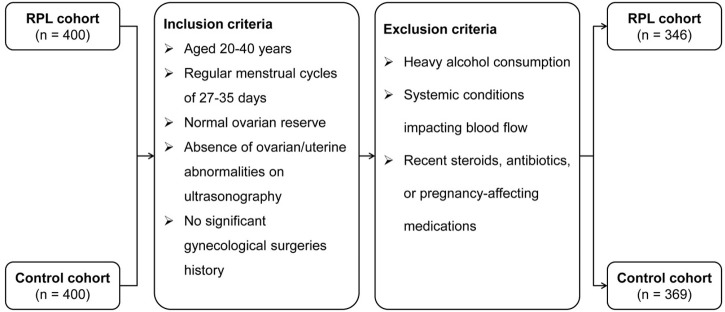
Participant selection flowchart for the RPL and control cohorts.

Inclusion criteria for both cohorts specified women aged 20–40 years with regular menstrual cycles (27–35 days) and normal ovarian reserve. Exclusion criteria included significant ovarian or uterine abnormalities, substantial alcohol consumption, systemic diseases affecting hemodynamic parameters, or recent use of medications like steroid hormones or antibiotics that could influence pregnancy outcomes. After screening, 346 uRPL patients and 369 controls were included in the study. Figure 1 illustrates the participant selection process.

### Ultrasound image acquisition

In the designated window of implantation (WOI), occurring 7–9 days post-ovulation (days 21–23 of the menstrual cycle), uniform transvaginal ultrasound scans were administered to all participants using the Resona R9T system (Shenzhen Mindray Corporation, Shenzhen, China). Two-dimensional grayscale images of the endometrium in a standard longitudinal section were systematically captured and archived for off-line analysis. Ultrasound data acquisition was performed by two experts, each with over a decade of experience in obstetric and gynecological ultrasonography.

### Tabular database establishment

A structured database was established from data collected with participants. This database incorporated demographic information and outcomes of previous pregnancies, including age, body mass index (BMI), and history of miscarriages. Key fertility indicators, such as levels of follicle-stimulating hormone (FSH), luteinizing hormone (LH), estradiol (E2), and antimüllerian hormone (AMH), were systematically recorded throughout the WOI. Additionally, ultrasonographic measurements, including endometrial thickness (EMT) and the vascular indices-namely, the pulsatility index (PI) and resistance index (RI) for both the uterine arteries (UA) and spiral arteries (SA)-were comprehensively integrated during this period.

### Data preparation prior to DL model development

**FIGURE 2:**
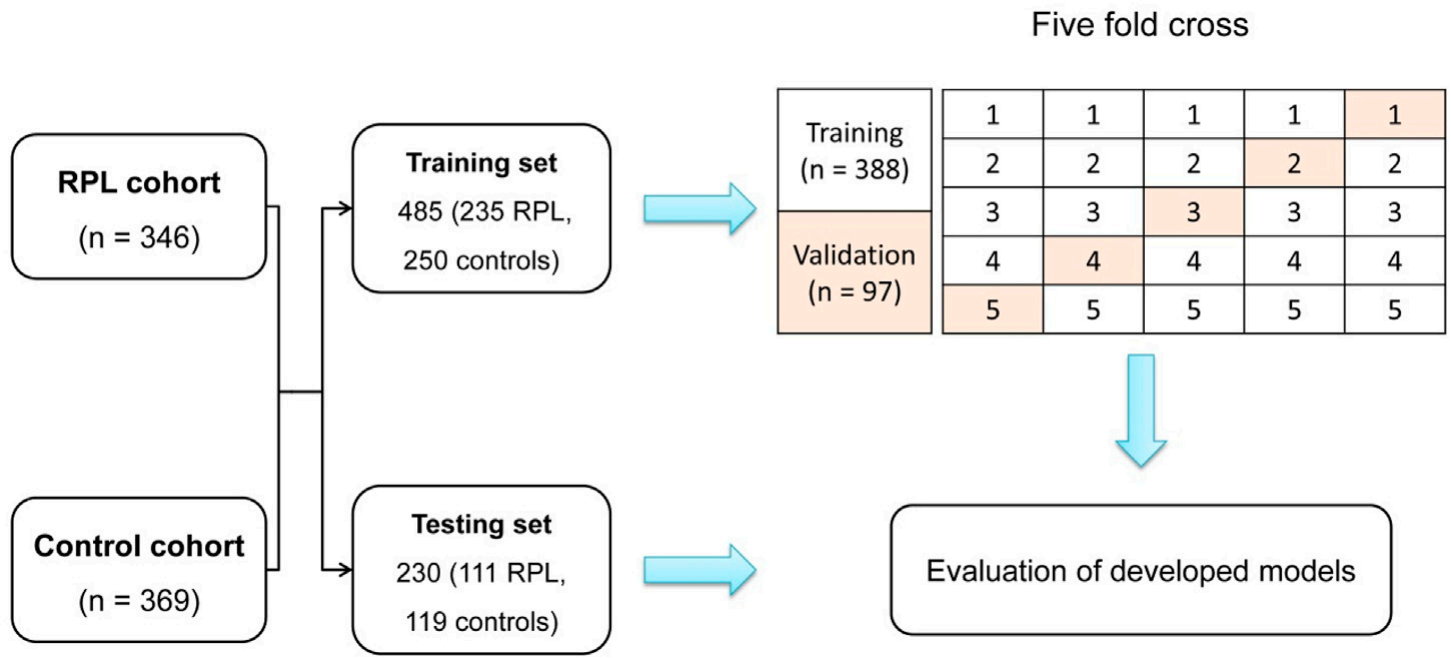
Depiction of participant stratification into training and testing sets for the training and evaluation of the DL model. The five-fold cross-validation method is employed, where subsets are sequentially designated for validation, ensuring a robust evaluation across diverse dataset partitions.

We included a total of 715 participants, consisting of 346 uRPL patients and 369 controls. The participants were divided into two datasets: a training set consisting of 485 individuals (235 with RPL and 250 controls) for model development, and a testing set comprising 230 cases (111 RPL cases and 119 controls) for evaluating the model’s performance. A stratified five-fold cross-validation was employed, where the training set was divided into five segments, each serving as a validation subset sequentially (Figure 2). The performance of the model in each fold was evaluated, and the iteration displaying the highest area under the receiver operating characteristic curve (AUC) during the validation phase was selected as the final model for further testing.

### Image pre-processing and data augmentation

Before incorporation into the neural network frameworks, central endometrial regions were extracted from ultrasound images and rescaled to 224 × 224 pixels using bilinear interpolation to optimize computational efficiency. Image preprocessing included Gaussian smoothing to reduce image noise and smooth pixel intensity variations, enhancing the model’s ability to detect relevant features by minimizing the impact of irrelevant high-frequency noise. Additionally, pixel standardization was applied to ensure consistent input quality for the neural network.

Data augmentation techniques were applied, including random horizontal and vertical flips, rotations within a range of ± 20°, and translations up to 15% of the image scale. These augmentations simulate common variations in ultrasound imaging, such as probe angle adjustments and patient positioning differences. By introducing these variations, the model is trained to recognize more robust and generalizable features, thereby minimizing overfitting and enhancing its accuracy on previously unseen data.

### DL prediction model

**FIGURE 3:**
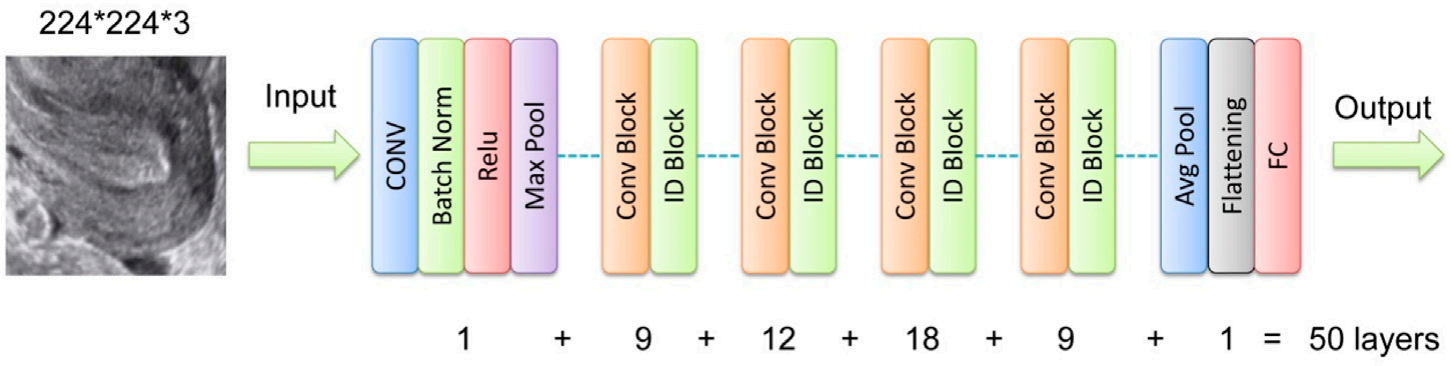
Network structure illustration for the DL model ResNet-50. Conv, convolutional layer; Batch Norm, batch normalization; ID, identity; FC, full connection.

Separate neural networks were trained to analyze image and tabular data, respectively. For the image data, the pre-trained ResNet-50 model was selected due to its robust feature extraction capabilities, particularly its ability to capture complex patterns in ultrasound images (He et al., 2016). The residual learning framework of ResNet-50 addresses the vanishing gradient problem, enabling high accuracy even in deep networks (Sharma and Singh, 2021). As illustrated in Figure 3, the initial final layer of the network, a softmax layer, was replaced with a sigmoid output layer specifically designed to estimate the RPL risk based on ultrasound imagery. The optimization of this network employed stochastic gradient descent (SGD) with a momentum of 0.9 and a binary cross-entropy loss function to enhance the precision of RPL risk prediction. The learning rate was initially set at 0.001, with a protocol to decrease it by a factor of 0.1 upon the plateauing of validation loss across ten epochs. Training proceeded for 10 epochs with a batch size of 64, balancing the need for precision with computational efficiency.

For tabular data, TabNet, a neural network framework optimized for handling high-dimensional clinical datasets, was chosen. Its attention mechanism enhances interpretability and efficiently models non-linear relationships, functioning similarly to a DL-based decision tree (Arik and Pfister, 2021). This architecture was selected for its stability, reliability, and proven effectiveness in clinical prediction tasks (Oba et al., 2021). The contribution of each feature within the tabular dataset to the model’s outcome predictions was quantitatively evaluated. A heatmap was generated to visualize feature importance, which played a critical role in improving the overall predictive accuracy of the model. Fine-tuning of the TabNet model involved the adjustment of several hyperparameters: a learning rate set at 0.1, utilization of the Adagrad optimizer for adaptive rate modifications, a cap of 200 epochs to facilitate model convergence, a patience setting of 50 to mitigate overfitting, and a batch size of 16 to streamline training processes. A hold-out test set was utilized to assess the model’s accuracy throughout the training phase. Both DL models were developed using the TensorFlow framework and Python 3.11.

The fusion model was constructed by integrating the outputs of the ResNet-50 and TabNet models to leverage both radiological and tabular data. Sigmoid outputs from ResNet-50 and TabNet were each converted into probabilistic scores representing RPL risk. These scores were then combined using a logistic regression (LR) model, where the scores from both models served as independent variables. The LR model generated a final probabilistic score for RPL risk by effectively integrating complementary information from both data sources. This integration is visualized in a nomogram, demonstrating the improvement in model accuracy achieved by combining the scores for RPL risk stratification.

### Evaluation and comparison of predictive models

Both the radiological and tabular data-derived DL models employed a sigmoid output to generate probabilistic scores indicative of the likelihood of RPL presence. Scores close to 1 suggest a higher risk, while those near 0 indicate normal fertility, with a threshold set at 0.5. To assess the effectiveness of our proposed model, we conducted comparative studies with other state-of-the-art architectures. For radiological data, we compared our model with DL architectures such as VGG-16 (Ritahani et al., 2024), DenseNet-121 (Chhabra and Kumar, 2024), and Inception-V3 (KIM, 2022). For tabular data, we included comparisons with traditional machine learning (ML) models such as LR, Support Vector Machines (SVM), and Random Forest (RF). Each model was configured and trained using the recommended hyperparameters specified in their respective official resource. A consistent training protocol was applied across all models for general optimization processes. Subsequently, all models were evaluated on the same testing cohort, ensuring that comparisons were conducted fairly and focusing on the inherent differences between the models’ architectures.

To evaluate the contributions of each data modality to the overall model performance and their impact on RPL risk prediction, we conducted an ablation study comparing the radiological DL model, the tabular DL model, and the fusion model integrating both data types. Evaluated on the same testing cohort, the concordance between model predictions and actual classifications was used to assess each model’s performance, focusing on metrics such as discriminatory capability, calibration, and clinical utility, thereby providing a comprehensive evaluation of how each modality contributes to the prediction process.

### Statistical analysis

Differences between the training and testing sets were evaluated using the Chi-square test, independent-sample *t*-test, or the Mann–Whitney *U* test, depending on the characteristics of the data. Predictive scores from the fusion model were obtained through logistic regression, incorporating scores from the radiological and tabular DL models. Model performance was assessed via receiver operating characteristic (ROC) curve analysis, with discrimination capability quantified by AUC, which underwent comparison through Delong’s test. Calibration curve analysis and the Brier score (BS) were employed to examine the goodness of fit for each model. Decision curve analysis (DCA) was utilized to assess the net benefits at various threshold probabilities, determining the models’ clinical applicability. Further, the evaluation of the model’s practical utility involved the analysis of key performance indicators, such as accuracy, precision, recall, and the F1 score, with a particular focus on the comparative model experiments and ablation studies conducted within the testing cohort. Analyses were conducted using Python version 3.11, with a *p*-value below 0.05 indicating statistical significance.

## Result

### Participant clinical characteristics

In the study involving 346 participants identified as experiencing RPL, the distribution of miscarriage events was observed as follows: 58.3% had encountered two miscarriages, 30.6% had three, and 11.1% reported four or more miscarriages. These individuals were systematically allocated into training and testing datasets for model development and performance assessment. Comparative evaluation of demographic, clinical, and ultrasonographic indices, as presented in Table 1, indicated no significant differences in baseline characteristics between the datasets (all *P* values >0.05), ensuring an unbiased foundation for analysis.

**TABLE 1:** Comparative analysis of demographic, clinical, and ultrasonographic characteristics between training and testing datasets.

Indicators	Training set (n = 485)	Testing set (n = 230)	Statistical values	*P* value
Age, year	33 (30–35)	33 (30–35)	1.026^a^	0.338
BMI, kg/m^2^	21.24 ± 3.73	21.17 ± 3.83	0.693^b^	0.528
Proportion of RPL, n (%)	235 (48.8%)	111 (48.3%)	0.002^c^	0.962
FSH, IU/L	7.36 (6.83–8.25)	7.53 (6.25–8.73)	0.527^a^	0.524
LH, IU/L	6.37 (5.45–7.22)	6.94 (5.24–7.23)	0.068^a^	0.934
E_2_, pg/mL	34.6 (29.2–39.4)	34.8 (29.6–39.7)	0.086^a^	0.914
AMH, ng/mL	1.44 ± 0.45	1.46 ± 0.47	0.545^b^	0.564
EMT, mm	8.95 ± 1.78	8.83 ± 1.85	0.263	0.828
SAPI	0.94 (0.85–1.12)	0.95 (0.84–1.16)	0.446^b^	0.675
SARI	0.55 ± 0.04	0.53 ± 0.56	0.735^b^	0.434
UAPI	2.14 ± 0.22	2.15 ± 0.23	0.096^b^	0.935
UARI	0.82 (0.81–0.83)	0.82 (0.79–0.83)	0.075^a^	0.936

^a^ For Mann–Whitney *U* test.

^b^ For independent-sample *t*-test.

^c^ For Chi-square test.

### Development of predictive models

Through univariate and multivariate LR analyses, we examined the association between clinical data from the training set and the likelihood of RPL. As illustrated in Table 2, the initial univariate analysis identified significant correlations between RPL risk and several factors, including age, SAPI, SARI, UAPI, and UARI. Subsequent multivariate regression analysis further refined these results, establishing age, SAPI, and SARI as independent predictors of elevated RPL risk. These variables were then used to construct a LR model for linear prediction.

**TABLE 2:** Univariate and multivariate logistic regression analysis of clinical indicators predictive of RPL risk.

Variable	Univariate logistic regression			Multivariate logistic regression		
	*P* value	*OR*	95% *CI*	*P* value	*OR*	95% *CI*
Age	<0.001	1.231	1.157–1.310	<0.001	1.242	1.161–1.328
BMI	0.210	0.967	0.917–1.019			
FSH	0.461	0.955	0.843–1.080			
LH	0.597	1.050	0.876–1.258			
E_2_	0.804	1.004	0.974–1.035			
AMH	0.159	0.735	0.479–1.128			
EMT	0.057	0.907	0.820–1.003			
SAPI	<0.001	1.461*	1.275–1.674	<0.001	1.534*	1.324–1.776
SARI	<0.001	2.171*	1.495–3.153	<0.001	2.288*	1.511–3.466
UAPI	0.031	1.103*	1.009–1.206	0.506	1.034*	0.936–1.143
UARI	0.015	1.909*	1.137–3.206	0.123	1.571*	0.885–2.792

The OR values represented by * is the elevated risk per 0.1-unit increment.

**FIGURE 4:**
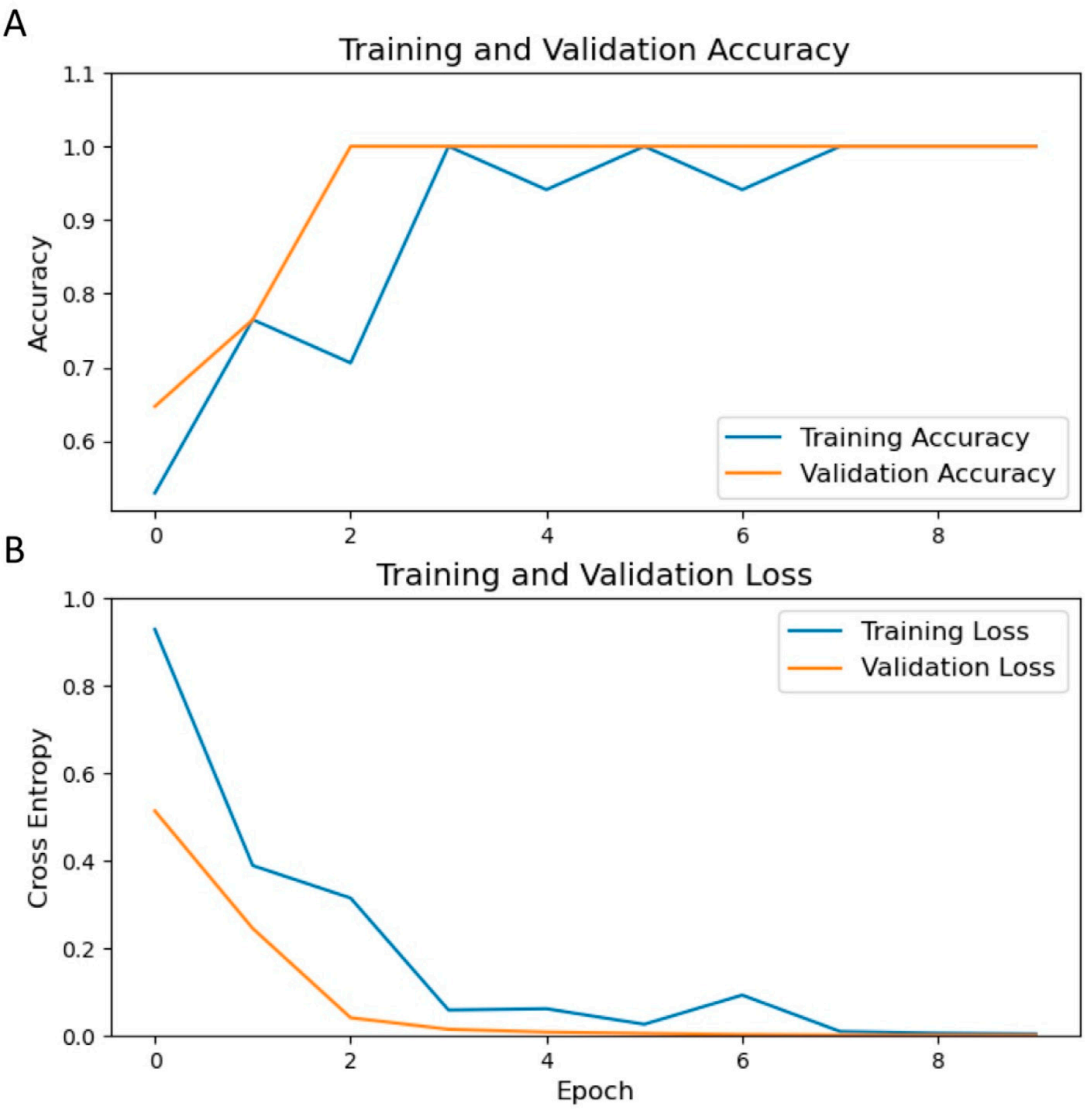
Trends in training and validation metrics over epochs. **(A)** displays the trajectory of accuracy for the training (blue) and validation (orange) sets, while **(B)** delineates the corresponding loss. An observable enhancement in accuracy and a decrease in loss are demonstrated throughout the training epochs.

The ResNet-50 architecture was employed for transfer learning, where ultrasound images of the endometrium from 235 RPL patients and 250 controls within the training set were utilized for model development. The customized radiological DL model, modified by replacing its final layer with a sigmoid output, was used to accurately estimate the risk of RPL. Optimization of the model was conducted over ten epochs. As depicted in Figure 4, significant enhancements in model accuracy were observed during the training phase. These enhancements were accompanied by reductions in loss across both the training and validation datasets, demonstrating effective learning and optimization processes.

**FIGURE 5:**
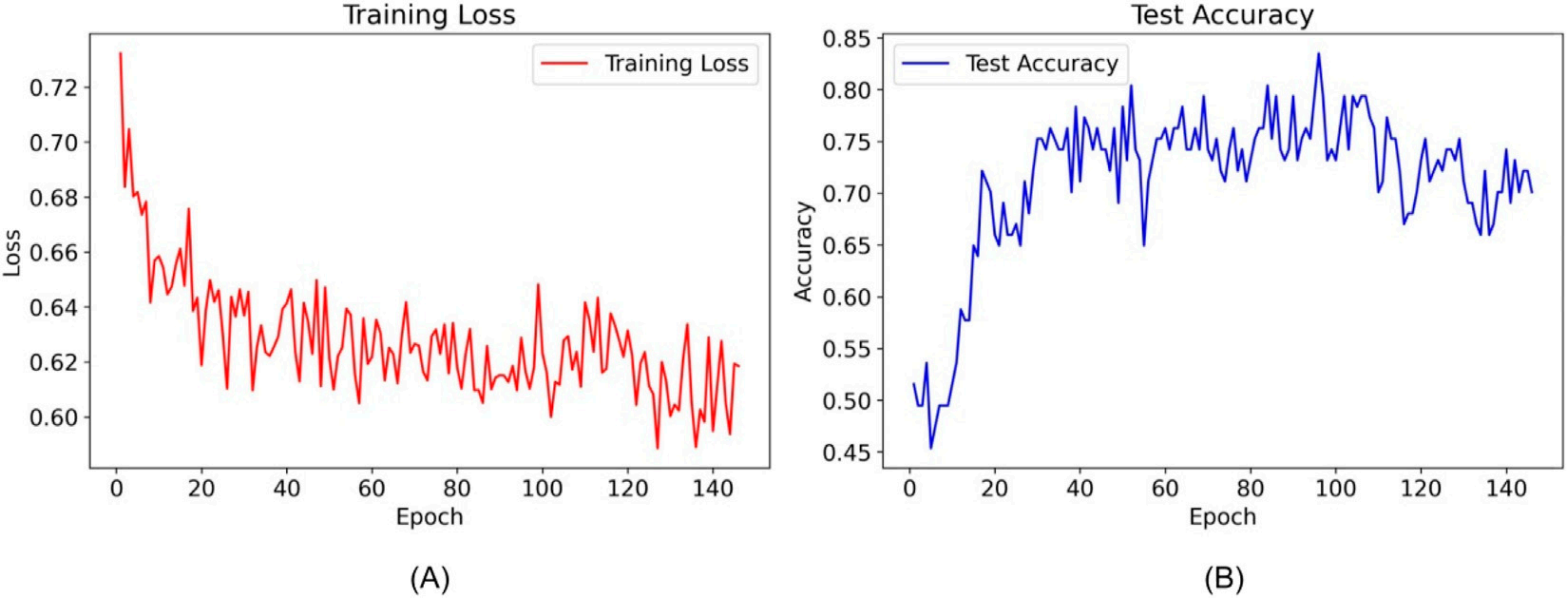
Training loss and test accuracy of the TabNet model over epochs. **(A)** shows the decrease in training loss, while **(B)** illustrates fluctuations in test accuracy, with epoch 95 marking the implementation of early stopping due to optimized model performance.

Utilizing the TabNet architecture, the tabular DL model was trained with clinical datasets to predict the risk of RPL. By epoch 95, an optimal test accuracy of 83.51% was achieved, triggering the early stopping mechanism as no further performance improvements were observed. This epoch was thus identified as the most effective in model performance. Graphical depictions of the training progression, illustrating accuracy and loss metrics, substantiated the numerical efficacy of the model as shown in Figure 5.

**FIGURE 6:**
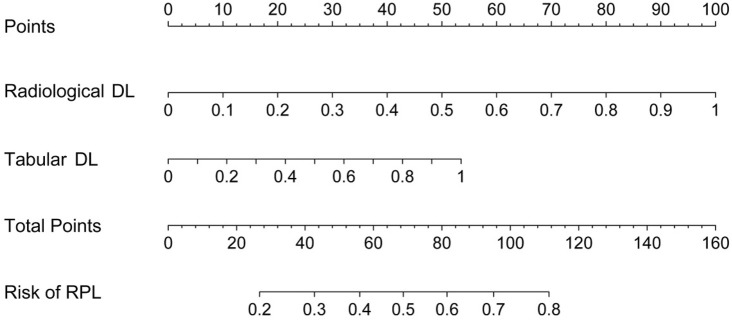
Nomogram for predicting RPL risk integrating outputs from radiological and tabular DL models.

The outputs from both the radiological DL model (ResNet-50) and the tabular DL model (TabNet) were converted into probabilistic scores representing the risk of RPL. These scores were subsequently integrated into an LR model to generate a final probabilistic score for RPL risk. By combining radiological and clinical data, this fusion model provided a more comprehensive risk assessment. The final integrated prediction is presented in a nomogram (Figure 6), demonstrating the improved capacity of the model to predict RPL risk through the integration of multiple data sources.

### Model comparison and ablation study results

**FIGURE 7:**
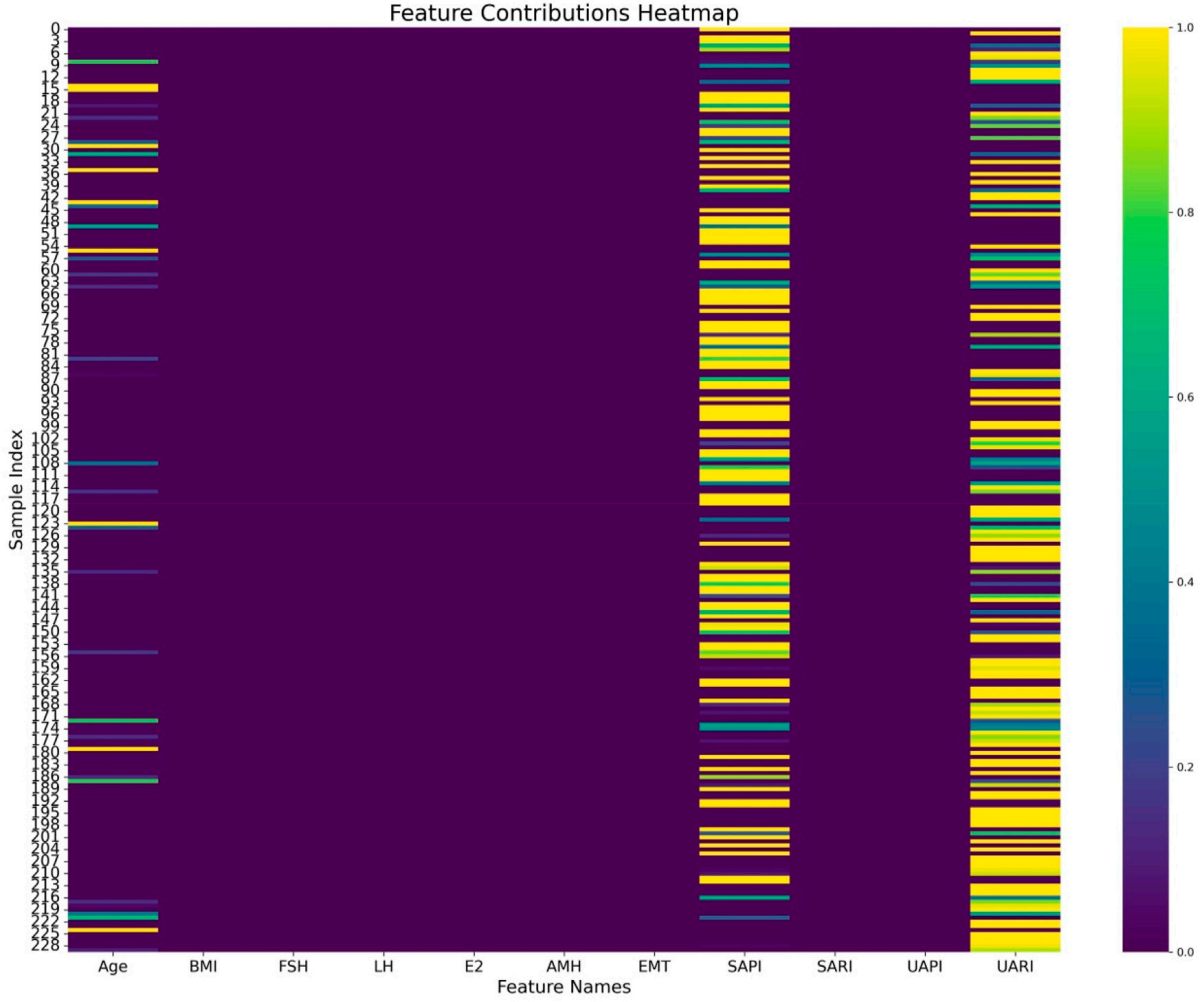
Heatmap visualization of feature contributions to the Tabular DL model, highlighting age, SAPI, and UARI as key factors in predicting high Ki-67 expression.

The comparative analysis of different DL and ML models on radiological and tabular data, as presented in Table 3, emphasizes the superior performance of the proposed ResNet-50 model for radiological data and the TabNet model for tabular data. Among radiological models, the ResNet-50 architecture demonstrated the highest overall performance, with an accuracy of 0.739 and the lowest BS of 0.175, reflecting both its strong predictive capability and good calibration. Considering the clinical context of RPL, where minimizing missed diagnoses (false negatives) is critical, ResNet-50 achieved the highest recall value of 0.748, demonstrating its heightened sensitivity in identifying high-risk patients. Similarly, the TabNet model outperformed traditional models such as LR, SVM, and RF in tabular data analysis, achieving an accuracy of 0.717 and a BS of 0.197. Its recall of 0.685 further confirmed its applicability in clinical risk stratification. The heatmap analysis (Figure 7) revealed that age, SAPI, and UARI made significant contributions to the predictive accuracy of the Tabular DL model, indicating a discrepancy in predictive variables between the Tabular DL and LR models. This discrepancy suggests that the nonlinear DL architecture, TabNet, is more suitable for predicting clinical outcomes.

**TABLE 3:** Comparative performance of different models on radiological and tabular data.

Model	Accuracy	Precision	Recall	F1 score	BS
Radiological data					
VGG-16	0.703	0.633	0.690	0.660	0.217
DenseNet-121	0.715	0.725	0.715	0.720	0.193
Inception-V3	0.710	0.702	0.704	0.703	0.187
ResNet-50 (Ours)	0.739	0.722	0.748	0.735	0.175
Tabular data					
LR	0.615	0.672	0.477	0.558	0.307
SVM	0.670	0.691	0.660	0.675	0.255
RF	0.682	0.664	0.680	0.672	0.231
TabNet (Ours)	0.717	0.717	0.685	0.700	0.197

**FIGURE 8:**
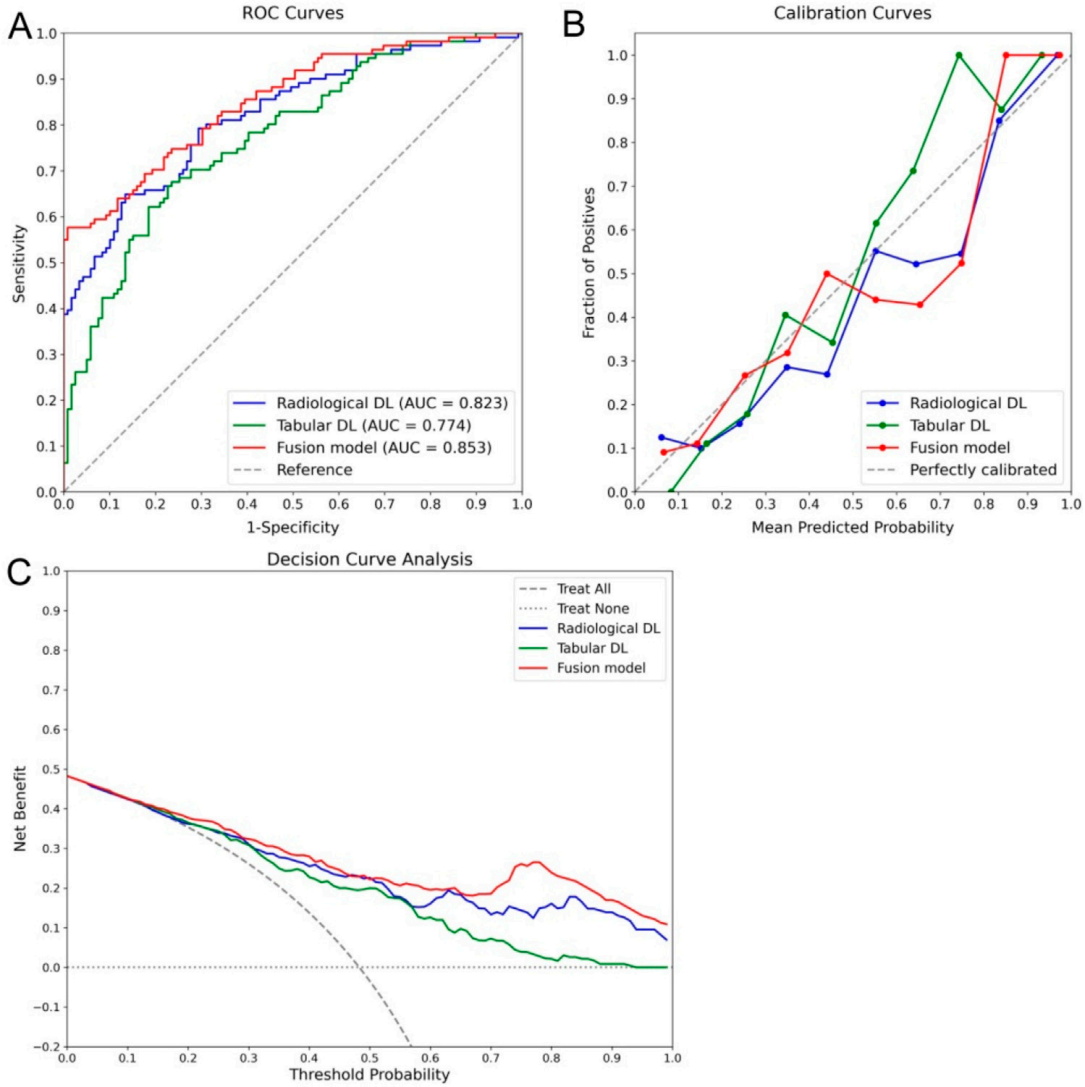
Ablation study results comparing the performance of radiological DL, tabular DL, and fusion models for RPL risk prediction. The fusion model (red) demonstrated the best overall performance across all metrics, including the highest AUC of 0.853 in the ROC curve **(A)**, superior calibration **(B)**, and the greatest clinical utility in the DCA **(C)**. Additionally, the radiological DL model (blue) consistently outperformed the tabular DL model (green), highlighting its more significant contribution to the overall performance of the fusion model.

In the ablation study, as shown in Table 4, the individual contributions of the radiological and tabular data modalities, as well as their integration within the fusion model, were assessed. The results demonstrated that the fusion model achieved the best overall performance, with an accuracy of 0.743 and the highest recall of 0.802, outperforming both the radiological DL-only and tabular DL-only models. This indicates the added value of integrating multimodal data to capture RPL cases. The fusion model also demonstrated improved calibration with the lowest BS (0.156), underscoring its potential as a reliable and clinically relevant tool for RPL risk prediction. Further analysis revealed that the radiological DL model surpassed the tabular DL model across all metrics, proving its greater contribution to the fusion model and highlighting the enhanced capability of DL networks based on ultrasonographic endometrial imagery for RPL risk prediction. These findings are further supported by the ROC curves, calibration curves, and DCA presented in Figure 8, where the fusion model achieved the highest AUC (0.853) and demonstrated superior calibration and clinical utility compared to the single-modality models. Additionally, the radiological DL-only model consistently outperformed the tabular DL-only model across all metrics.

**TABLE 4:** Results of the ablation study comparing radiological DL, tabular DL, and fusion models for RPL risk prediction.

Model	Accuracy	Precision	Recall	F1 score	BS
Radiological DL only	0.739	0.722	0.748	0.735	0.175
Tabular DL only	0.717	0.717	0.685	0.700	0.197
Fusion model	0.743	0.706	0.802	0.751	0.156

## Discussion

In advancing routine screening for RPL risk, this study demonstrates the integration of DL technologies with endometrial ultrasound imaging and clinical data to improve ER assessment. By employing pre-trained ResNet-50 for imaging and TabNet for clinical data, this approach achieved higher accuracy in dataset analysis, surpassing traditional methods during testing. The model comparison experiment indicated that the trained TabNet model outperformed the linear RF model and other traditional ML models when analyzing identical clinical data. The ablation study further revealed that the radiological DL model outperformed the tabular DL model. The fusion model, which integrates radiological and tabular data, proved to be the superior predictive tool in this study. This non-invasive and reliable method effectively differentiates high-risk RPL patients from healthy individuals, enabling more precise therapeutic interventions. Characterized by its integrated use of routinely collected data, the study leverages the power of DL to refine ER evaluations, offering novel insights and methodologies for RPL risk management.

### AI-based ultrasound models for ER assessment

The critical role of ER in implantation success and pregnancy initiation is highlighted by its facilitation of embryonic attachment and development (Miravet-Valenciano et al., 2015). Identification of the WOI, a critical period for optimal embryo acceptance by the endometrium, is essential through ER assessment (Lessey, 2000). Disturbances in ER during the WOI are correlated with RPL, emphasizing a compromised implantation process as a significant factor in pregnancy maintenance failure (Patel and Lessey, 2011). The process of decidualization, involving the transformation of endometrial fibroblasts into decidual cells, is critical for concluding the implantation window (Krieg et al., 2013). This transformation facilitates the endometrium’s ability to recognize and eliminate non-viable embryos (Goto et al., 2023). Deficiencies in decidualization may escalate the risks of implantation delays, inadequate embryo evaluation, and early placental dysfunction (Coulam et al., 2020). Thus, the emphasis on the WOI for ER assessment is critical in examining the link between ER anomalies and RPL, underscoring the importance of precise and timely ER evaluations to mitigate RPL risks.

Currently, AI-based models utilizing ultrasound for ER assessment represent a cutting-edge approach, with recent studies demonstrating superior accuracy compared to traditional methods, such as EMT measurements, particularly in predicting embryo implantation success in assisted reproductive technology (ART) cycles (Fjeldstad et al., 2024). Ultrasound, as the primary modality for obstetric imaging, is being explored through advanced techniques like ultrasound elastography and radiomics to provide more detailed insights into tissue properties and structural features relevant to ER evaluation. Research has shown that ultrasound elastography improves the precision of pregnancy outcome predictions in ART cycles (Li et al., 2024), while DL models applied to ultrasound radiomics have achieved high accuracy in predicting implantation success after frozen embryo transfer (Liang et al., 2023). Furthermore, the integration of three-dimensional ultrasound data with AI has been shown to outperform traditional markers such as EMT in assessing ER (Wang et al., 2022; Ricardo et al., 2024). These studies have achieved ER assessment accuracies similar to those found in our research, underscoring the potential of AI-enhanced ultrasound in this area. However, most of these studies have focused on ART, leaving a significant gap in applying AI-enhanced ultrasound models for ER evaluation in RPL. Accurate ER assessment is crucial not only for identifying women at higher risk of RPL but also for improving endometrial conditions in RPL patients to enhance pregnancy success rates (Haouzi et al., 2014). This study addresses this gap by applying AI-based ultrasound models specifically to RPL, improving both the accuracy and clinical relevance of ER evaluation in this context.

### Justification for model selection in RPL risk prediction

The selection of ResNet-50 and TabNet in this study was based on their proven ability to handle the complexity of medical imaging and clinical data. ResNet-50, with its deep residual learning framework, was chosen over other CNN architectures like VGGNet and DenseNet due to its capacity to address the vanishing gradient problem, enabling deeper training without loss of performance (Xu et al., 2023). This feature is critical in medical imaging, where subtle patterns in ultrasound images must be detected for accurate prediction of ER and RPL risk (Nagaraju and Rao, 2022). In this study, ResNet-50 demonstrated the highest accuracy among the radiological models, achieving 0.739, along with a recall of 0.748. This emphasizes the model’s superior sensitivity in identifying RPL risk, which is crucial for minimizing missed diagnoses in clinical practice.

TabNet was selected for tabular data analysis because it better handles complex, non-linear relationships that are common in high-dimensional clinical datasets (Yingze et al., 2024). Its attention mechanism enables the model to focus on the most relevant features, allowing for more accurate prediction of RPL risk by capturing interactions between various clinical factors (Wang and Sun, 2022; Park et al., 2023). In the tabular data analysis, TabNet outperformed traditional models such as LR, SVM, and RF, with an accuracy of 0.717 and a recall of 0.685. This highlights the model’s ability to effectively identify at-risk individuals while maintaining a high sensitivity, which is particularly important in clinical settings where minimizing false negatives is a priority.

### Advantages of DL models in RPL prediction

In the model comparison experiment, the TabNet model outperformed the linear LR model, likely because the DL model can capture complex, non-linear relationships among variables that linear models struggle to address. Notably, UARI emerged as a critical predictor in the TabNet model but was not retained in the LR model, possibly due to issues with multicollinearity. In traditional linear models, UARI may lose statistical significance during multivariate analysis due to overlapping effects with other variables. In contrast, the DL model, such as TabNet, can better handle and utilize these intricate, interdependent relationships between predictors, which linear models often fail to do due to their sensitivity to multicollinearity and limited capacity to model non-linear interactions (Han et al., 2017).

Further, the superiority of the radiological DL model over the tabular DL model can be attributed to the rich, high-dimensional data contained within medical images. Imaging offers a comprehensive view of the physiological state, capturing nuances that tabular data alone may not fully represent. This aligns with existing literature that demonstrates the enhanced capability of imaging-based DL models in medical diagnostics, where the spatial and morphological information within images provides critical insights into disease states and risks (Panayides et al., 2020). The integration of visual patterns through CNNs allows for a more comprehensive assessment of ER and the subsequent risk of RPL.

### Contributions of the proposed model

The proposed fusion model demonstrates enhanced predictive capability by integrating radiological and tabular data, leveraging the radiological model’s ability to detect subtle image features and the tabular model’s strength in interpreting clinical data. This integration strategy is particularly beneficial in a multimodal setting, where the physiological and clinical aspects of patient data complement each other, offering a more reliable and nuanced approach to RPL risk stratification. The combination of ResNet-50 and TabNet, as evidenced by the ablation study, achieved the highest performance, with an accuracy of 0.743 and a BS of 0.156. This approach allows for a more detailed and accurate risk assessment compared to single-modality models. The model’s AUC of 0.853 further highlights its superior discriminatory ability, marking a significant improvement over existing RPL prediction models (Bashiri et al., 2022; Sugiura-Ogasawara et al., 2009). Given the challenges associated with the relatively low incidence of RPL (Youssef et al., 2022), the focus is often on minimizing the oversight of high-risk cases. The fusion model’s recall score of 0.8 demonstrates its ability to accurately identify 80% of high-risk cases, representing a substantial advancement in RPL risk stratification.

### Limitation

Despite the demonstrated efficacy of the proposed DL approach in assessing RPL risk, the study is constrained by several limitations. First, the sample size is relatively small, which is partly due to the low incidence rate of uRPL in the general population, making large-scale data collection a challenging endeavor. While the current sample size provides valuable insights, future studies with larger, multi-center cohorts are needed to validate the model and improve its statistical power and external validity. Second, the ultrasound images used for model development was sourced from a single manufacturer and clinical center, which may compromise the model’s generalizability across different clinical settings. Standardization of imaging protocols and the inclusion of multiple imaging modalities (e.g., color Doppler ultrasound, elastography) in future studies could further enhance the robustness of the model. Furthermore, while the model demonstrates potential, its direct clinical applicability remains a challenge, and the development of a user-friendly interface for clinical integration is a critical step for ensuring its practical utility in routine RPL screening. Additionally, the ResNet-50 and TabNet models used in this study, while effective, are pre-trained models not specifically designed for medical applications. While they have been successfully adapted for medical use, there is potential for the development of DL models more specifically tailored to the unique characteristics of medical imaging and clinical data. Future research could explore the use of purpose-built DL architectures, which may offer improved performance for medical applications.

## Conclusion

This study reveals the efficacy of a DL methodology in refining RPL risk assessment by integrating radiological and clinical data through advanced models such as ResNet-50 and TabNet. This fusion model, utilizing DL techniques, demonstrates superior accuracy and efficiency over conventional diagnostic methods. Notably, this approach is based on routinely collected data, allowing for easier large-scale implementation in clinical practice. The method can be readily adopted by clinicians, promoting broader and more effective risk stratification for RPL.

## Data Availability

The original contributions presented in the study are included in the article/supplementary material, further inquiries can be directed to the corresponding author.
